# Fifteen new earthworm mitogenomes shed new light on phylogeny within the *Pheretima* complex

**DOI:** 10.1038/srep20096

**Published:** 2016-02-01

**Authors:** Liangliang Zhang, Pierfrancesco Sechi, Minglong Yuan, Jibao Jiang, Yan Dong, Jiangping Qiu

**Affiliations:** 1 School of Agriculture and Biology, Shanghai Jiao Tong University, Shanghai, China; 2 Institute of Ecosystem Study (ISE), Italian National Research Council, Sassari, Italy; 3 College of Pastoral Agricultural Science and Technology, Lanzhou University, Gansu, China

## Abstract

The Pheretima complex within the Megascolecidae family is a major earthworm group. Recently, the systematic status of the Pheretima complex based on morphology was challenged by molecular studies. In this study, we carry out the first comparative mitogenomic study in oligochaetes. The mitogenomes of 15 earthworm species were sequenced and compared with other 9 available earthworm mitogenomes, with the main aim to explore their phylogenetic relationships and test different analytical approaches on phylogeny reconstruction. The general earthworm mitogenomic features revealed to be conservative: all genes encoded on the same strand, all the protein coding loci shared the same initiation codon (ATG), and tRNA genes showed conserved structures. The *Drawida japonica* mitogenome displayed the highest A + T content, reversed AT/GC-skews and the highest genetic diversity. Genetic distances among protein coding genes displayed their maximum and minimum interspecific values in the *ATP8* and *CO1* genes, respectively. The 22 tRNAs showed variable substitution patterns between the considered earthworm mitogenomes. The inclusion of rRNAs positively increased phylogenetic support. Furthermore, we tested different trimming tools for alignment improvement. Our analyses rejected reciprocal monophyly among *Amynthas* and *Metaphire* and indicated that the two genera should be systematically classified into one.

Earthworms (Annelida: Oligochaeta) are arguably the most important global players of the soil biota in terms of soil formation, impact on soil structure and fertility^1^. They were a major interest of Charles Darwin during his lifetime. His investigations on the subject led him to state that earthworms are likely the most important animal in the history of the world^2^. The understanding of the earthworms’ impact on global ecology increased vastly and steadily since the days of Darwin, giving depth to his original statement. Interestingly, it was recently discovered that, although they are clearly the primary ecosystem engineers of soil environments and beneficial to soil fertility^3^, earthworms even contribute to net soil greenhouse-gas emissions^4^.

*Pheretima* complex is one of the largest groups within the Megascolecidae earthworm family, with 12 genera, including approximately 930 valid species^5^,^6^. *Amynthas* and *Metaphire* are two pheretimoid genera widely distributed in East Asia. In China, these two genera are dominant, as their presence sums up to the 81.9% of the total earthworm fauna^7^. They have similar morphological characteristics; the only difference between them concerns the presence of copulatory pouches in the male pore areas, as these structures are present in *Metaphire* but missing in *Amynthas*^8^,^9^. However, the definition of copulatory pouch is now controversial, largely due to different criteria^10^. Recent phylogenetic analyses based on the mitochondrial COI and 16S genes, did not support the reciprocal monophyly of both *Amynthas* and *Metaphire*^6^,^11^,^12^. However, molecular works so far were based on only a few loci, and they were poorly resolved. To deal with this challenge, better approaches would involve the use of multiple concatenated genes longer than 2000 bp, in order to provide enough phylogenetic information^13^.

The rise in availability of genomic resources and data in the last decades is leading to an increasing number of studies using complete mitochondrial genomes, in order to investigate phylogenetic relationships among taxa. Mitogenomes have been proven powerful in resolving phylogenetic relationships across a wide range of metazoans^14^,^15^. In addition to be more informative than single genes, complete mitogenomes enable the additional analysis of evolutionary significant genome features, such as gene content and gene order^16^. Up to now, more than 5,000 mitochondrial genome sequences of metazoans have been deposited in the public databases (http://www.ncbi.nlm.nih.gov, last accessed August 22, 2015). Surprisingly, despite the important role that earthworms play as key organisms in terrestrial ecosystems, very few earthworm mitogenomes were published since the first report on the mitogenome of *Lumbricus terrestris*^17^. To date, only 9 earthworm mitogenomes are available in GenBank. Among them, six species were sequenced in our previous studies (Table 1). This number is clearly negligible when compared to the wealth of earthworm diversity. More earthworm mitogenomes are needed in order to deepen our understanding of evolutionary relationships within this important animal group at the genomic level.

Here we determined the complete mitochondrial sequences of 15 earthworm species, and analyzed them with the 9 ones previously available in Genebank, with the aim to explore the phylogenetic signal of complete mitochondrial genomes in earthworms. We investigated the influence of different approaches to phylogeny reconstruction. In addition, we tested the hypothesis that *Amynthas* and *Metaphire* are not monophyletic separate clades.

## Materials and Methods

### Sample collection and DNA extraction

Specimen information is shown in Table 1. All experimental protocols were approved by the Animal Ethics Committee of Shanghai Jiao Tong University School of Agriculture and Biology. All specimens were anesthetized in a 10% ethanol solution and then preserved in 90% ethanol and stored at 4 °C until DNA extraction. Whole genomic DNA was obtained from fresh tissue by dissection of individual adult earthworms and extracted following the protocol of Mollusc DNA Kit (OMEGA E.Z.N.A.TM).

### PCR and sequencing

Primers designed to amplify generally conserved regions of earthworm mtDNA were used to obtain short fragments from *CO1*, *CO2*, *CO3*, *Cytb*, *ND5*, *ND4*, *16 S* and *ND1* (Supplementary Table S1). Specific primers (Supplementary Table S2) were designed based on these conserved regions and used to amplify the remainder mtDNA sequence in several PCR reactions. The PCR reactions were carried out with LA Taq polymerase for 35 cycles at 94 °C for 30 s, and annealed at 50 °C for 30 s, followed by extension at 72 °C for 1 min per 1 kb. The final MgCl_2_ concentration in the PCR reaction was 2.0 mmol/L. PCR products were cloned with the pGEM-T vector (Promega, USA) and then sequenced, or sequenced directly by Sanger sequencing, using an ABI 3730 automatic sequencer. The fragments obtained were assembled with the software DNAstar and adjusted manually to generate complete mitochondrial DNA sequences.

### Sequence analysis

The online softwares MITOS^18^ and DOGMA^19^ were used for gene annotation. The online tools ARWEN^20^ and tRNAscan-SE^21^ were used to confirm tRNA annotation results. The boundaries of the predicted genes were finally confirmed by sequence comparisons with the reported earthworms mitogenomes. Comparison of nucleotide identity was made using the CG View Comparison Tool (CCT). The base composition and pairwise genetic distances of both PCGs (Protein Coding Genes) and earthworm species were analyzed with MEGA 5^22^. AT and GC skews were estimated with the formula AT-skew = (A−T)/(A + T) and GC-skew = (G−C)/(G + C)^23^.

### Phylogenetic analysis

Besides the mitochondrial genomes of 24 earthworms, 2 species from Hirudinea (*Whitmania laevis* and *Whitmania pigra*) were selected as outgroup. For phylogenetic analyses, the 37 mitochondrial genes were separately aligned by the MUSCLE algorithm, and DNA sequences of PCGs were translated to protein in MEGA5. The individual alignments were then concatenated using the software SequenceMatrix v1.7.6^24^. In order to evaluate the effect of data partitioning and incorporation of RNAs on phylogeny, several datasets were generated as follows to test the effect of the optimizing schemes (Table 2):

1. PCGs, nucleotide sequences of 13 Protein Coding Genes (PCG);
2. PRO, protein sequence of 13 PCGs (PRO);
3. PCGs + rRNAs, nucleotide sequences of 13 PCGs plus rRNAs (PR);
4. PCGs + tRNAs, nucleotide sequences of 13 PCGs plus tRNAs (PT);
5. PCGs + rRNAs + tRNAs (PRT);
6. rRNAs;
7. tRNAs;

In order to remove unreliably aligned regions within the datasets, we used Gblocks (gb) and trimAl (tri) in the datasets (1), (2) and (3) to identify the conserved regions with default parameters. For the remainder datasets, only trimAl was used.

All datasets were partitioned by gene, with the exception of tRNAs dataset which was combined as one partition due to their small size. Two phylogenetic approaches were applied, including Maximum Likelihood (ML) using raxmlGUI^25^ and Bayesian phylogenies using Mrbayes3.2.1^26^. Evolutionary models selections for each dataset were carried out using MrModeltest implemented in MrMTgui for nucleotide sequences, and ModelGenerator v0.85^27^ for protein sequences. Since the MtZoa evolutionary model^28^ for amino acid data was not available on ModelGenerator, we evaluated tree topologies based on MtZoa and Mtrev + I + G (the best-fit model according to ModelGenerator) separately, and MtZoa was chosen as the best-fit model because it provided better support values and less computational time. For ML analyses, node support was calculated via rapid bootstrapping and “autoMR” bootstopping under GTRCAT. For BI analyses, two independent runs of 10^7^ generations (until average standard deviation of split frequencies < 0.01) were conducted simultaneously, sampling every 1000 generations and discarding 25% of the initial trees as burnin. The remaining sampled trees were used to estimate the 50% majority rule consensus tree.

In addition, to avoid arbitrary and subjective defining data blocks, we also used PartitionFinder 1.1.1^29^ to objectively assess the best partition scheme in PRTtri (PCGs + rRNAs + tRNAs after trimAl) and PRtri (PCGs + rRNAs after trimAl) datasets. For the PRtri dataset, the input alignment was predefined to 41 data blocks, corresponding to the codon position of each of the 13 PCGs, plus the two rRNA genes. We used the Bayesian information criterion (BIC) and the “greedy” algorithm with branch lengths estimated as “linked” to search for the best-fit scheme. However, subsequent phylogenetic analyses had no effect on tree topology and slight effect on nodal support compared to manual partition schemes.

## Results

### Characteristics of earthworm Mitochondrial Genomes

**Figure 1:**
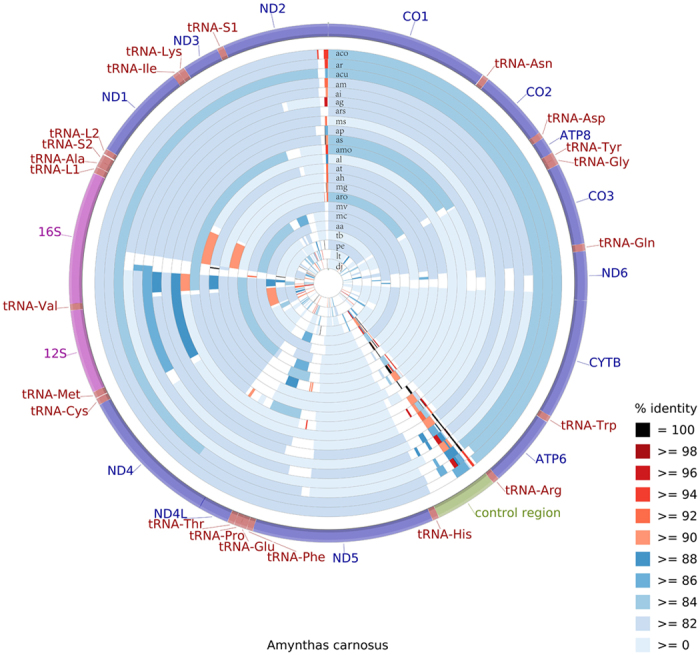
Graphical map of the BLAST results showing nucleotide identity between *A. carnosus* mitogenome and 23 other earthworm species listed in Table 1. CCT arranges BLAST result in an order where sequence that is most similar to the reference (*A. carnosus*) is placed closer to the outer edge of the map. Species are abbreviated as following: *aa, A. aspergillus; ac, A. carnosus; aco, A. corticis; acu, A. cucullatus; ag, A. gracilis; ah, A. hupeiensis; ai, A. instabilis; al, A. longisiphonus; am, A. moniliatus; amo, A. morrisi; ap, A. pectiniferus; ar, A. redactus_sp.nov; aro, A. robustus; ars, A. rongshuiensis_sp.nov; as, A. spatiosus_sp.nov; at, A. triastriatus; mc, M. californica; mg, M. guillelmi; ms, M. schmardae; mv, M. vulgaris; pe, P. excavatus; tb, T. birmanicus; lt, L. terrestris, dj, D. japonica*.

The characteristics of the 15 newly sequenced earthworm mitogenomes were summarized and compared with the 9 previously published earthworm mitogenomes. All the twenty-four earthworm mitogenomes contain the typical 37 genes present in metazoans^30^. As already observed in all the annelid species studied so far, all genes are transcribed from the same strand. Furthermore, these genomes show that the mitochondrial gene order is always conserved within oligochaetes. The earthworm mitogenomes display slight size variation, ranging from 14,998 bp in *D. japonica* to 15,188 bp in *A. pectiniferus* (Table 1). For *D. japonica*, however, we failed to obtain the non-coding region between *trnR* and *trnH* (assumed to be the A + T-rich region). The CCT BLAST map shows the sequence identity between *A. carnosus* and other worm species, varying between 63–82% (Fig. 1). The control region sequences of these earthworms are highly divergent. All analyzed mitogenomes are relatively uniform in the overall nucleotide composition (A + T content between 61.6–69.7%). Interestingly, *L. terrestris* (Lumbricidae) is at the low end and *D. japonica* (Moniligastridae) is at the high end of the range (Table 1), leaving worms within Megascolecidae in the middle. The nucleotide bias was also evident when analyzing base skewness. The earthworms within Megascolecidae are slightly A-skewed (0.03–0.07), while *L.terrestris* and *D. japonica* pertaining to other families, exhibit negative AT-skews (−0.03 and −0.15). Moreover, The GC-skew of *D. japonica* is 0.04, whereas other worms show negative GC-skews.

### Protein-coding genes

Earthworm mtDNA typically contains 13 Protein coding genes (PCGs). In all species, ATG is the unique start codon; this is common in annelid mitogenomes, whereas most of metazoan mt genomes use also alternative start codons^31^,^32^. The PCGs are terminated by either the complete (TAA or TAG) or incomplete stop codons (TA-, T-), which can completed to TAA by polyadenylation after transcription^33^.

**Figure 2:**
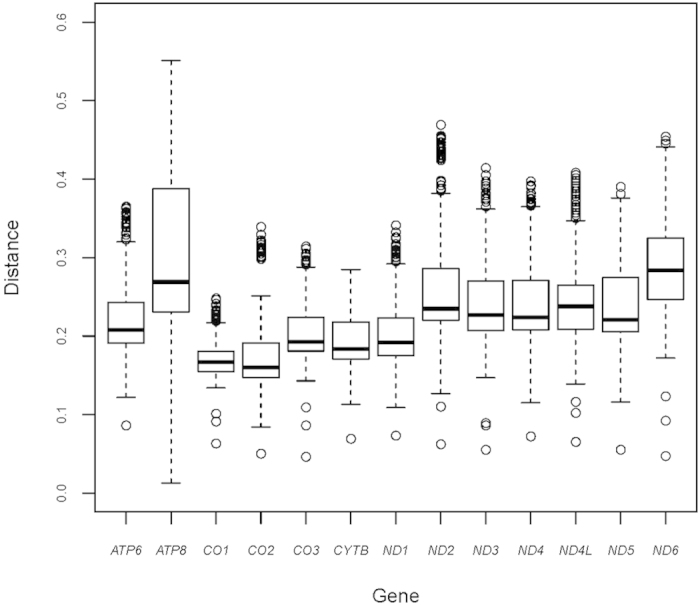
Genetic distances of individual genes. Each boxplot represents P distance for 13 individual genes in 24 earthworm species. Lower horizontal bar, non-outlier smallest observation; lower edge of rectangle, 25 percentile; central bar within rectangle, median; upper edge of rectangle, 75 percentile; upper horizontal bar, non-outlier largest observation; open circle, outlier.

The pairwise genetic distances within 24 species based on single PCG are shown in Fig. 2. *ATP8* is the least conserved gene (averaged 0.303, range 0.013–0.551). *CO1* is the most conserved gene (averaged 0.172, range 0.063–0.248), and is therefore a useful marker inferring phylogenetic relationships at higher taxonomic levels.

Pairwise genetic distances based on concatenated 13 PCGs were estimated for three different taxonomic levels. Distances among *Pheretima* complex reveal uniform variation (the first 20 species, mean 0.194), ranging from 0.062 between *M. vulgaris* and *M. guillelmi* to 0.220 between *M. californica* and *A. longisiphonus* (Supplementary Fig. S1, Supplementary Table S3). Interestingly, the *CO1* divergence between *M. vulgaris* and *M. guillelmi* is also 0.063, nearly identical to the whole PCGs level. Our results suggest that these two species are closely related, as they display the lowest *CO1* interspecific distance, which is far less than the mean interspecific value of 0.172 in our dataset, and also the mean interspecific P-distance in Megascolecidae (18.66%) as demonstrated by Chang^34^. Within the family Megascolecidae, *P. excavatus* and *T. birmanicus* show relatively higher variation; and beyond Megascolecidae, *L. terrestris* and *D. japonica* reveal high sequence divergence compared to other worms, and there is extremely high nucleotide diversity in *D. japonica* (averaged 0.334, range 0.322–0.346).

### Comparison of tRNA genes

**Figure 3:**
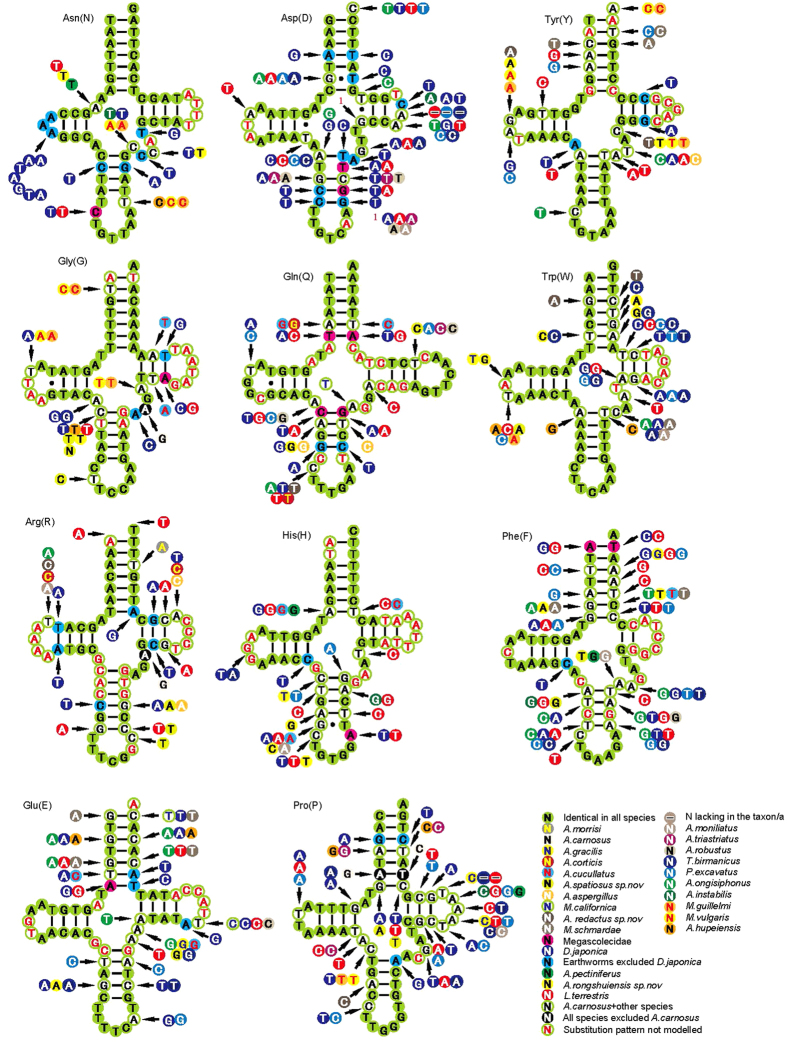
Secondary structure of tRNA families (trnN-trnP) in earthworm mtDNAs. The nucleotide substitution pattern for each tRNA family was modeled using as reference the structure determined for *A. carnosus*.

**Figure 4:**
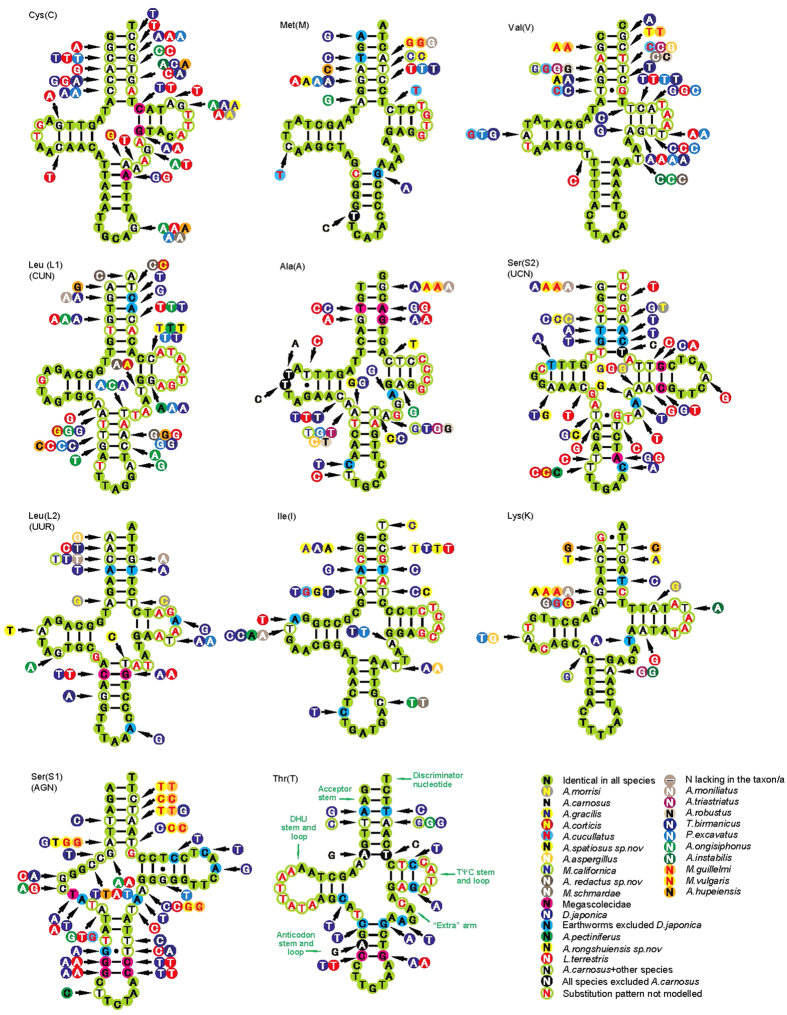
Secondary structure of tRNA families (trnC-trnT) in earthworm mtDNAs. The nucleotide substitution pattern for each tRNA family was modeled using as reference the structure determined for *A. carnosus*.

The results of comparative analyses on secondary structures of earthworm tRNAs are provided in Figs 3 and 4. The postulated tRNA cloverleaf structures always contain 7 bp in the aminoacyl stem, 2–5 bp in the TψC stem, 4–6 bp in the anticodon stem, and and 3–4 bp in the DHU stem. Among the 22 tRNAs, only *trnS1* does not exhibit the common cloverleaf structure, due to the absence of DHU stem. The lack of D stems in *trnS1* is a widespread feature of mitochondrial tRNA genes^35^,^36^. The percent of identical nucleotides (%INUC) is calculated based on alignments of orthologous sequences (Supplementary Table S4). *trnM, trnN, trnK and trnI* show the highest levels of nucleotide conservation (%INUC > 60), followed by tRNAs at large in 50 < %INUC < 60. *TrnF, trnL1* and *trnP* are between 40 and 50, while *trnS2* is the least conserved tRNA (%INUC < 40).

The most conserved tRNAs show nucleotide substitutions largely restricted to TΨC loops and acceptor arms (Figs 3 and 4). Acceptor stems show 0–4 fully compensatory base changes (cbcs) (e.g., G-C vs. A-T in *trnM*) and/or hemi-cbc (e.g., A-T vs. G-T in *trnM*). In most tRNAs, it was impossible to model the substitution patterns in the TΨC loop due to a high level of variation among orthologous sequences. Notablely, the D stem, which is assumed to act as a recognition site for aminoacyl-tRNA synthetase^37^, keeps the highest conservation with a few sustitutions.

Cbcs and hemi-cbcs are restricted to individual species or characterized taxa at a higher taxonomic level (family/order), as reported in insects^38^. We also find the similar substitution patterns in our study. An example of the first type is the C-G pair in the *trnN* acceptor arm of *D. japonica* is mirrored by T-A in all other earthworms (Fig. 3). In addition, the DHU loop in the *trnN* of *D. japonica* is distinct from that of any other species. An example of a full cbc characterizing a unique family is the T-A pair found in the acceptor stem of *trnAs* of the family Megascolecidae, while the other two species *D. japonica* and *L. terrestris*, not belonging to Megascolecidae, exhibit the C-G pair. Note that in *M. vulgaris* and *M. guillelmi*, not only the same substitutions patterns are present in compensatory changes of stems (e.g. *trnM*), but also in base changes of loops and extra arms (e.g. *trnY*), indicating they are closely related species. Figures 3 and 4 depict more examples.

Furthermore, some tRNAs present mismatched pairs in stems (e.g., T-T in the anticodon stem of *trnN*; C-C in the TψC stem of *trnQ*; G-G in the aminoacyl stem of *trnA*). These mismatches are common in annelids^36^,^39^ and it has been proposed that they may be corrected through editing processes^40^ or that they could represent unusual pairings^41^.

### Methodological effects of various approaches

**Figure 5:**
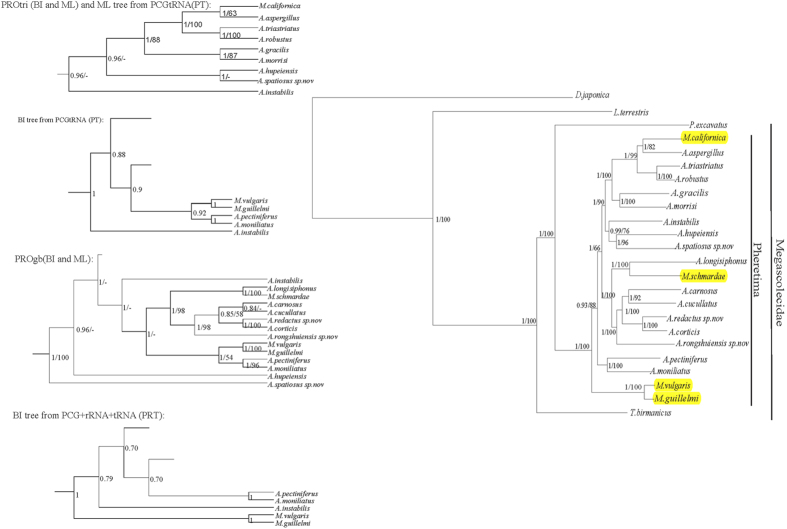
The consensus tree is shown on the right. It was inferred from the PRtri dataset (PCGs + rRNAs after trimming with the trimAl software). The internal branches within the Pheretima complex are too short to discern; thus, the amplified tree without the outgroup is presented. Support values are posterior probabilities from Bayesian inferences (PP) and likelihood values from ML analyses (BP). The datasets displayed on the left show different types of relationships, recovered either in Bayesian and Maximum likelihood analyses or only in Bayesian analyses. Species with yellow colour in the main tree belongs to genus Metaphire.

We have performed 20 independent phylogenetic analysis to test the influence of the optimizing schemes under two inference methods (BI and ML), different datasets types (DNA or protein, RNA inclusion/exclusion), and two alignment trimming tools (Gblocks or trimAl). Several datasets are generated in Table 2. In general, five different tree topologies were recovered (Fig. 5).

Nine phylogenetic analyses (BI and ML trees of PCGgb, PCGtri, PRgb, PRtri and ML tree from PRT) produce a consensus topology, and the tree topology of PRtri with high node support is presented in Fig. 5. Three analyses—BI and ML trees of PROtri, and ML tree of PT—recovered similar topologies to the consensus tree. Only a minor difference was detectable when these three topologies were compared to the consensus topology: in these datasets, *A.instabilis* located in a more basal position within the pheretima complex. BI and ML methods often converge on a single topology using the same dataset (PCGgb, PCGtri, PROgb, PROtri, PRgb and PRtri). The results of different analyses are presented in Supplementary Fig. S2.

For the different data treatments, we compared the effects of recoding PCG nucleotide sequences into amino acid sequences, inclusion of rRNA or tRNA genes with PCGs against PCGs alone (Fig. 5 and Supplementary Fig. S2). The PRO dataset fails to get a consensus tree, different basal taxa were recovered within Pheretima complex. Inclusion of rRNA genes has slight effect on BI tree topology but positive effect on nodal support in ML trees. This suggest rRNAs contribute positive signal to phylogenetic analyses. However, different relationships are recovered within Pheretima complex when tRNA genes are included. The single analyses based on rRNAs ant tRNAs perform poorly compared to the combined dataset, resulting in incongruent topologies from ML and BI inference methods and internal polytomy topologies (data not shown).

Three datasets (PCG, PRO and PR) were compared using these two trimming methods trimAl and gblocks. For the PCG and PR dataset, we observed an overall improvement of supported topologies using trimAl over gblocks. Furthermore, after gblocks trimming the PROgb dataset showed a totally different topology from the PROtri and remaining datasets, thus we considered it unsupported. Gblocks is over stringent especially when trimming rRNA, trimmed alignments are much shorter than the alignments after trimAl (1444 vs 1711 bp). We checked the less stringent parameters in Gblocks and yield 1973 bp, which may be better than its default setting. Collectivelly, trimAl outperformed Gblocks with default parameters in our datasets.

To avoid arbitrary and subjective defining data blocks, we also used PartitionFinder to find the best partitioning scheme. Unexpectedly, subsequent phylogenetic analyses had no effect on tree topology and slight effect on nodal support compared to manual partition schemes.

### Phylogeny

The consensus tree from PRtri (Fig. 5) shows that there is strong support for the monophyly of Megascolecidae and Pheretima complex (Both PP = 1.00, BS = 100). It is evident that *M. vulgaris* and *M. guillelmi* within the genus *Metaphire* cluster in a sister group and split early from other pheretimoids. Other two *Metaphire* species *M. californica* and *M. schmardae* scatter and mingle in the genus *Amynthas*; thus, the two genera are not reciprocally monophyletic.

## Discussion

In the present study, we sequenced 15 new earthworm mitochondrial genomes and we analyzed them together with the 9 ones already available in Genbank, including one incomplete mitogenome (*D. japonica*) from a previously unsampled family (Moniligastridae). The main non-coding region of the *D. japonica* genome, however, could not be retrieved. According to the sequence length reduction of *D. japonica* when compared to other earthworm species, and judging from the high AT content, the secondary structure, and the stretches of polyT in earthworm A + T-rich region, it is likely that a sequence of about 500 bp was omitted in our PCR amplification of the *D. japonica*, in spite of repeated amplification and sequencing efforts. This region likely contains regulatory secondary structures, several tandem repeats, and stable stem-loop structures; these features may hinder results of PCR and sequencing reactions, as previously reported for other annelids^36^,^39^,^42^,^43^,^44^. Interestingly, the *D. japonica* mitogenome displayed the highest A + T content (69.7%), AT/GC-skews reversal and the highest genetic diversity when compared to any other earthworm genomes. It was worth noting that the AT-skew and GC-skew of the *D. japonica* are precisely opposite when compared with other earthworms, which have a positive AT-skew and negative GC-skew. In insect mitogenomes, the reverse skewness may be caused by inversion of replication origin in the A + T-rich region^45^, but as the non-coding region for *D. japonica* could not be retrieved, the exact reason for its skewness reversal remains unknown. *D. japonica* is a member of Moniligastridae, which might explain its distinct mitochondrial genome features, as they retain primitive features (large-yolked eggs and single layered clitellum) typical of aquatic oligochaetes, presumably more primitive than any other earthworms^46^.

In this study, we tested different mitogenome phylogenies by implementing two inference methods (BI and ML), including combinations of rRNA or tRNA genes, and removing variable regions with Gblocks or trimAl softwares. In most instances (except the PT and PRT datasets), BI analyses recovered a more consistent tree topology across different datasets, and nodal support was remarkably high for the majority of nodes. It has been suggested in several studies that posterior probabilities overestimate the real support^47^,^48^. Thus, any conclusions drawn from the BI analysis alone should be considered with caution.

We further tested the effect of gene exclusion by comparing the combined analyses of PCGs + rRNA/tRNA with analyses of PCGs alone. RNAs are often discarded in phylogenetic analyses^49^,^50^,^51^. However, some studies concluded that the inclusion of these genes is beneficial, as it resulted in improved resolution and nodal support^52^,^53^. In our analyses, the inclusion of rRNA genes had positive effect on nodal support and in the congruence of topologies, especially in ML analyses. By contrast, inclusion of tRNAs resulted in more diverse and poorly supported phylogenetic relationships comparing with other datasets (Fig. 5 and Supplementary Fig. S2).

Removal of poorly aligned regions should greatly improve phylogeny reconstruction^54^. In order to improve alignments, dedicated trimming softwares have been developed, selecting blocks of conserved regions and removing poorly aligned or ambiguous positions. We tested the Gblocks and trimAl softwares^54^,^55^. Compared to Gblocks, trimAl is more recently developed and better fit to analyze large-scale data, as it has the possibility to automatically adjust the parameters to improve the phylogenetic signal-to-noise ratio^56^. In our analyses, trimAl outperformed Gblocks v0.91 when set to default parameters, resulting in an overall improvement of supported topologies. Therefore, trimAl seems to be a softer, good alternative.

Although some conflicting results are observed from different datasets and inference methods, our results all strongly support the monophyly of Megascolecidae and Pheretima complex (Fig. 5). Furthermore, all analyses consistently reject the monophyly of *Amynthas* and *Metaphire*. This hypothesis received also support by recent works based on individual genes^6^,^11^,^12^. In fact, the only diagnostic character between *Amynthas* and *Metaphire* is the presence/absence of copulatory pouches^10^. Considering their morphological similarity and our mitogenome phylogenetic evidence, we suggest that these two genera should be put into one genus. Indeed, the inclusion of extra species from other genera within the Pheretima complex is clearly needed to validate this taxonomic revision. Furthermore, as can be seen in Supplementary Fig. S3, branch lengths within the *Pheretima* complex are generally short, suggesting that pheretimoid worms diversity may have resulted from a relatively recent and rapid radiation.

## Conclusions

In this study, we sequenced and annotated the mitogenomes of fifteen pheretimoid earthworm species. This work includes the first comprehensive phylogenetic analysis of earthworms using mitogenomes. Our results showed that the general earthworm genomic features are conservative. Peculiarly, The *D. japonica* genome showed the highest A + T content (69.7%), reversed AT/GC-skews and highest genetic diversity compared to any other earthworm genomes. Our Phylogenetic results indicated that inclusion of rRNAs positively increase the nodal support of topologies. Our Phylogenetic analyses provided evidence that *Amynthas* and *Metaphire* are non-monophyletic separate clades; in view of their morphological similarity, these two genera should be put into one genus, and their current taxonomic classifications should be revised. This study provides insights into the evolution and phylogeny of chinese pheretimoid earthworms and related species, and it is the first study to investigate the deep evolutionary relationship between important earthworm clades. Our conclusions will be strengthened by the inclusion of extra species from other genera within the Pheretima complex and possibly other earthworm orders and families.

## Additional Information

**How to cite this article**: Zhang, L. *et al*. Fifteen new earthworm mitogenomes shed new light on phylogeny within the *Pheretima* complex. *Sci. Rep*. **6**, 20096; doi: 10.1038/srep20096 (2016).

## Supplementary Material

Supplementary Information

## Figures and Tables

**Table 1:** Characteristics of 24 eathworm Mitochondrial Genomes.

species	family	GenBank Accession	Genome Length (bp)	AT%	AT Skew	GCSkew	Reference	Location	GPS Coordinates
*L. terrestris*	Lumbricidae	NC_001673	14998	61.6	−0.03	−0.18	17		
*M. vulgaris*	Megascolecidae	NC_023836	15061	64.6	0.04	−0.15	57	Shanghai	N31.1477°E121.3613°
*A. aspergillus*	Megascolecidae	NC_025292	15115	63.0	0.06	−0.21	58	Guangdong	N23.1139°E113.3011°
*P. excavatus*	Megascolecidae	NC_009631	15083	64.5	0.04	−0.20	unpublished		
*T. birmanicus*	Megascolecidae	KF425518	15170	63.3	0.06	−0.20	59		
*M. californica*	Megascolecidae	KP688581	15147	64.1	0.05	−0.19	60	Shanghai	N31.0324°E121.4419°
*A. longisiphonus*	Megascolecidae	KM199289	15176	66.2	0.04	−0.15	60	Chongqing	N29.0146°E107.1394°
*A.corticis*	Megascolecidae	KM199290	15127	66.3	0.05	−0.17	60	Guangxi	N21.8471°E107.8887°
*A. gracilis*	Megascolecidae	KP688582	15161	65.5	0.05	−0.18	60	Guangxi	N21.4858°E107.5701°
*A. carnosus*	Megascolecidae	KT429008	15160	62.6	0.05	−0.16	This study	Shanghai	N31.1477°E121.3613°
*A. hupeiensis*	Megascolecidae	KT429009	15069	65.9	0.07	−0.19	This study	Shanghai	N30.9675°E121.0111°
*M. guillelmi*	Megascolecidae	KT429017	15174	65.3	0.03	−0.16	This study	Shanghai	N31.0324°E121.4419°
*A. pectiniferus*	Megascolecidae	KT429018	15188	66.2	0.06	−0.18	This study	Shanghai	N31.0324°E121.4419°
*A. morrisi*	Megascolecidae	KT429011	15026	65.4	0.04	−0.17	This study	Chongqing	N29.6042°E106.3947°
*A. robustus*	Megascolecidae	KT429019	15013	64.9	0.04	−0.17	This study	Guangxi	N24.1690°E110.2441°
*A. triastriatus*	Megascolecidae	KT429016	15160	65.3	0.04	−0.18	This study	Guangxi	N21.8471°E107.8887°
*A. instabilis*	Megascolecidae	KT429007	15159	64.9	0.06	−0.18	This study	Guangxi	N21.8452°E107.8872°
*M. schmardae*	Megascolecidae	KT429015	15156	66.7	0.03	−0.15	This study	Hunan	N27.2568°E112.7243°
*A. cucullatus*	Megascolecidae	KT429012	15122	64.8	0.05	−0.16	This study	Jiangxi	N28.0939°E117.0189°
*A. redactus sp.nov*	Megascolecidae	KT429010	15131	67.6	0.05	−0.18	This study	Hunan	N26.0078°E113.8866°
*A. moniliatus*	Megascolecidae	KT429020	15133	66.7	0.06	−0.18	This study	Hunan	N25.9771°E113.7163°
*A. spatiosus sp.nov*	Megascolecidae	KT429013	15152	66.2	0.05	−0.18	This study	Jiangxi	N28.1238°E116.9897°
*A. rongshuiensis sp.nov*	Megascolecidae	KT429014	15086	67.2	0.04	−0.17	This study	Guangxi	N25.2032°E108.6807°
*D. japonica*	Moniligastridae	KM199288	14646	69.7	−0.15	0.04	This study	Shanghai	N31.1477°E121.3613°

Abbreviation: *L. Lumbricus; P. Perionyx; T. Tonoscolex; M. Metaphire; A. Amynthas; D. Drawida*.

**Table 2:** Brief summary of the datasets.

Dataset name	Dataset size (bp)
PCGgb	10761
PCGtri	10857
PROgb	3472
PROtri	3599
PRgb	12069
PRtri	12568
PT	12053
PRT	13764
rRNA	1711
tRNA	1257

Abbreviation: gb, Gblocks; tri, TrimAl.
