# Supplementary material for: Fifteen new earthworm mitogenomes shed new light on phylogeny within the Pheretima complex
Source: Sci Rep. 2016 Feb 1;6:20096. doi: 10.1038/srep20096 (PMC4735579; doi:10.1038/srep20096)
Supplement: Supplementary Information [file srep20096-s1.pdf]

Fifteen new earthworm mitogenomes shed new light on phylogeny within the *Pheretima* complex

Liangliang Zhang[1], Pierfrancesco Sechi[2], Minglong Yuan[3], Jibao Jiang[1], Yan Dong[1] & Jiangping Qiu[1]*

Supplementary Figure S1. Genetic distances of individual species (Species are abbreviated as Fig 2)

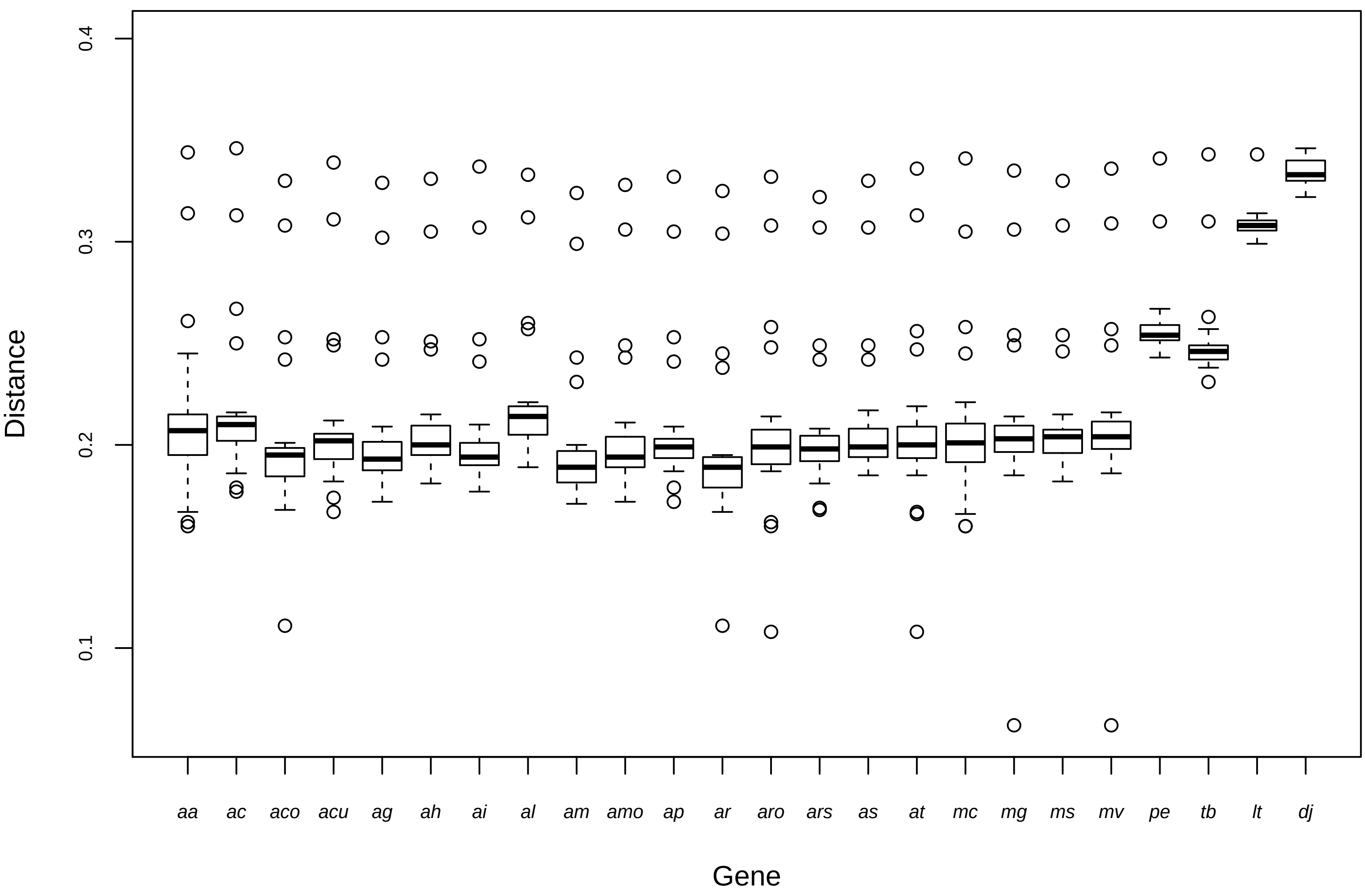

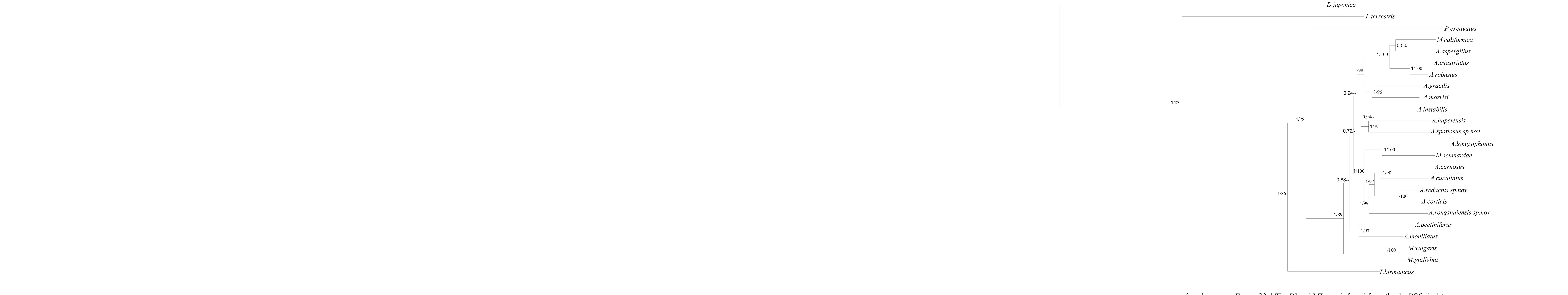

Supplementary Figure S2-1 The BI and ML tree inferred from the the PCGgb dataset

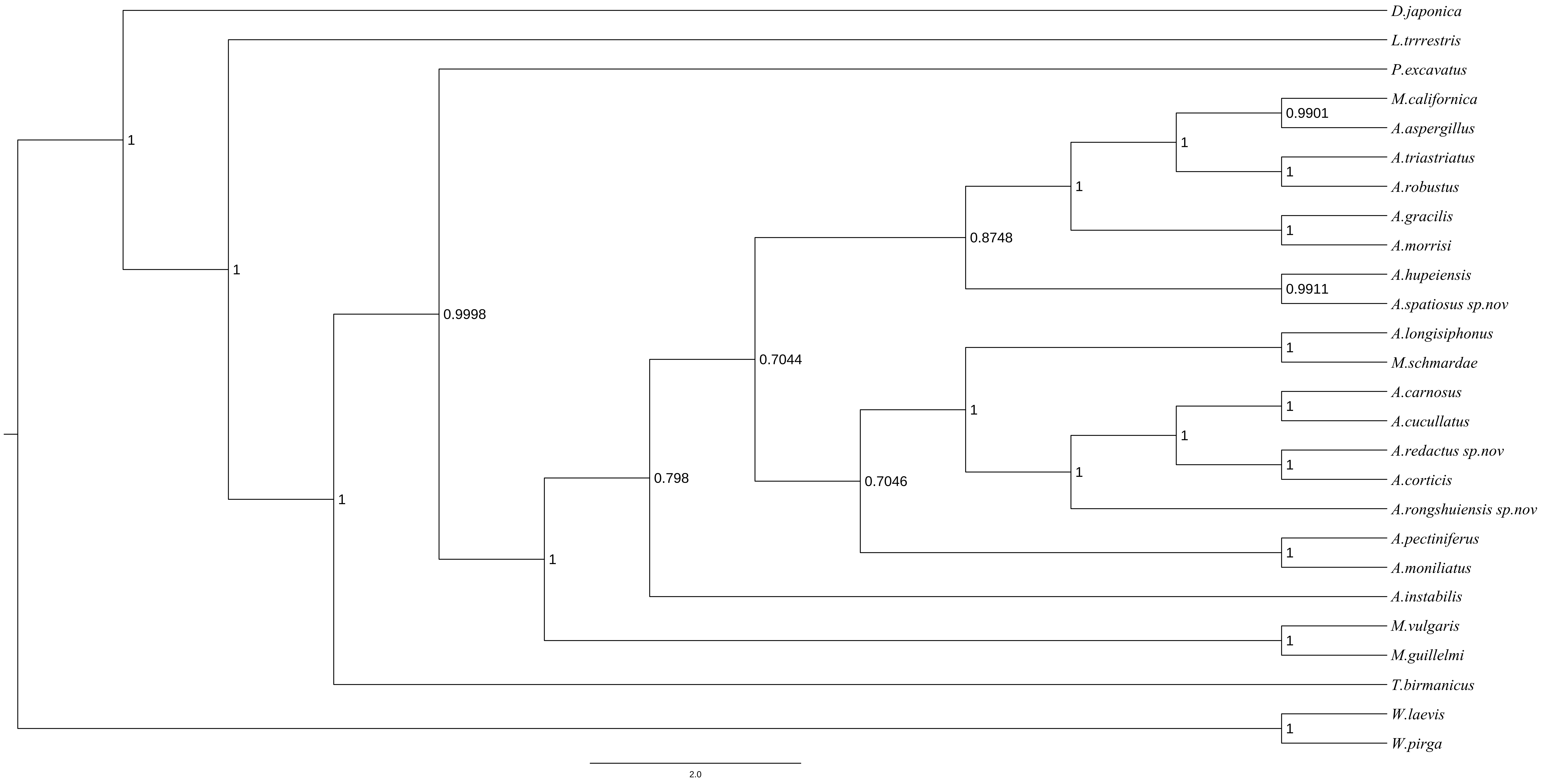

Supplementary Figure S2-2 The BI tree inferred from the the PRT dataset

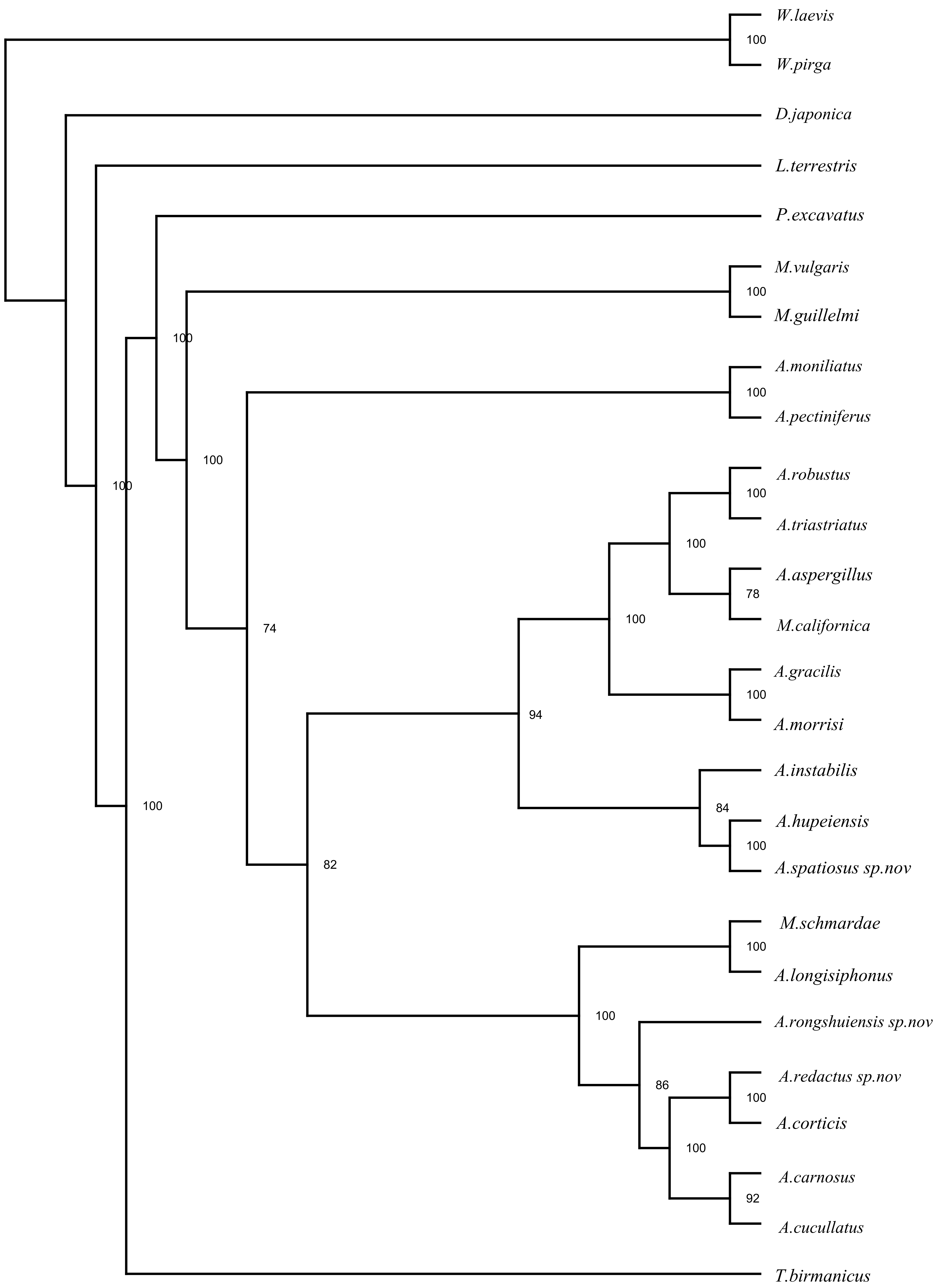

Supplementary Figure S2-3 The ML tree inferred from the the PRT dataset

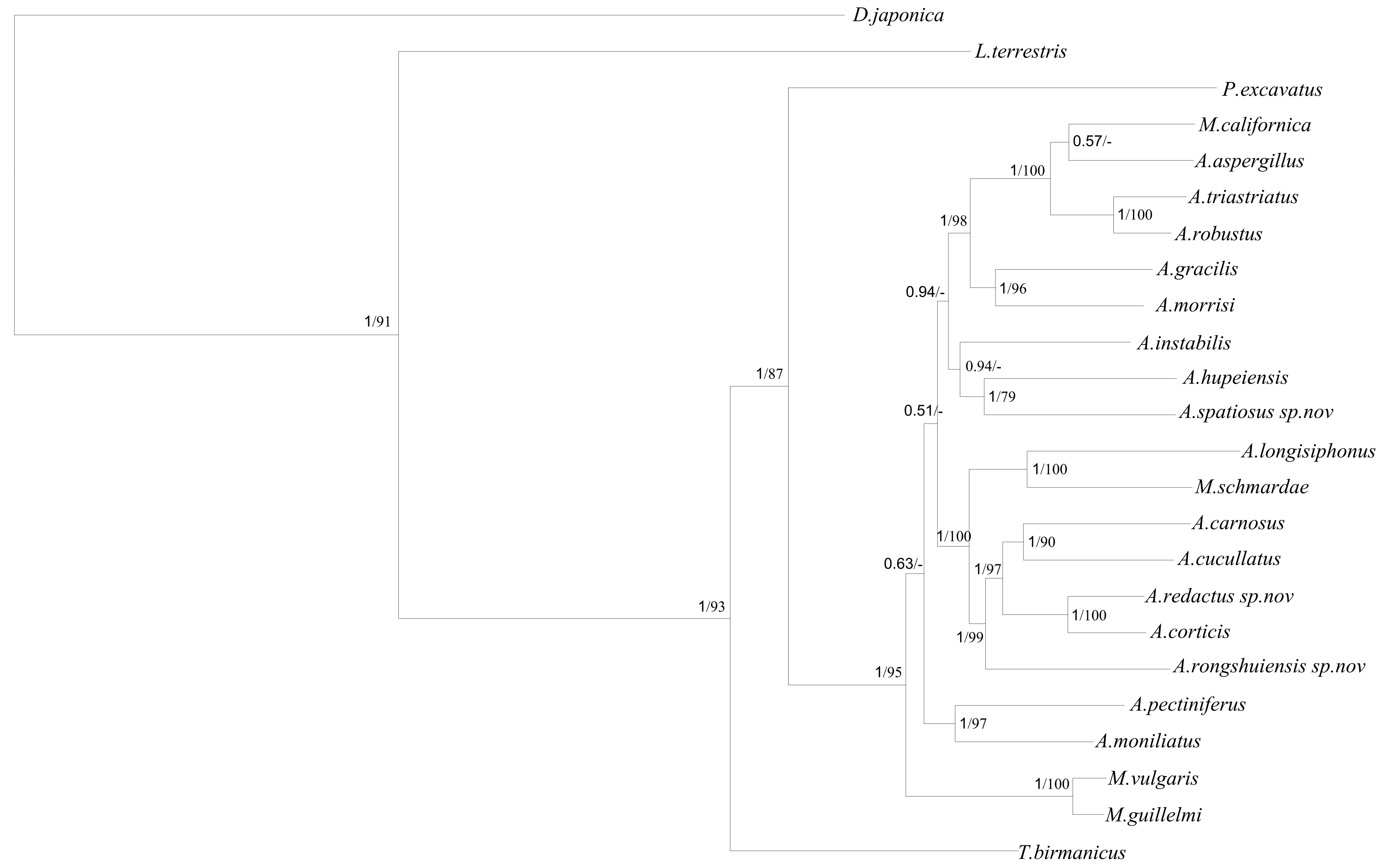

Supplementary Figure S2-4 The BI and ML tree inferred from the the PRgb dataset

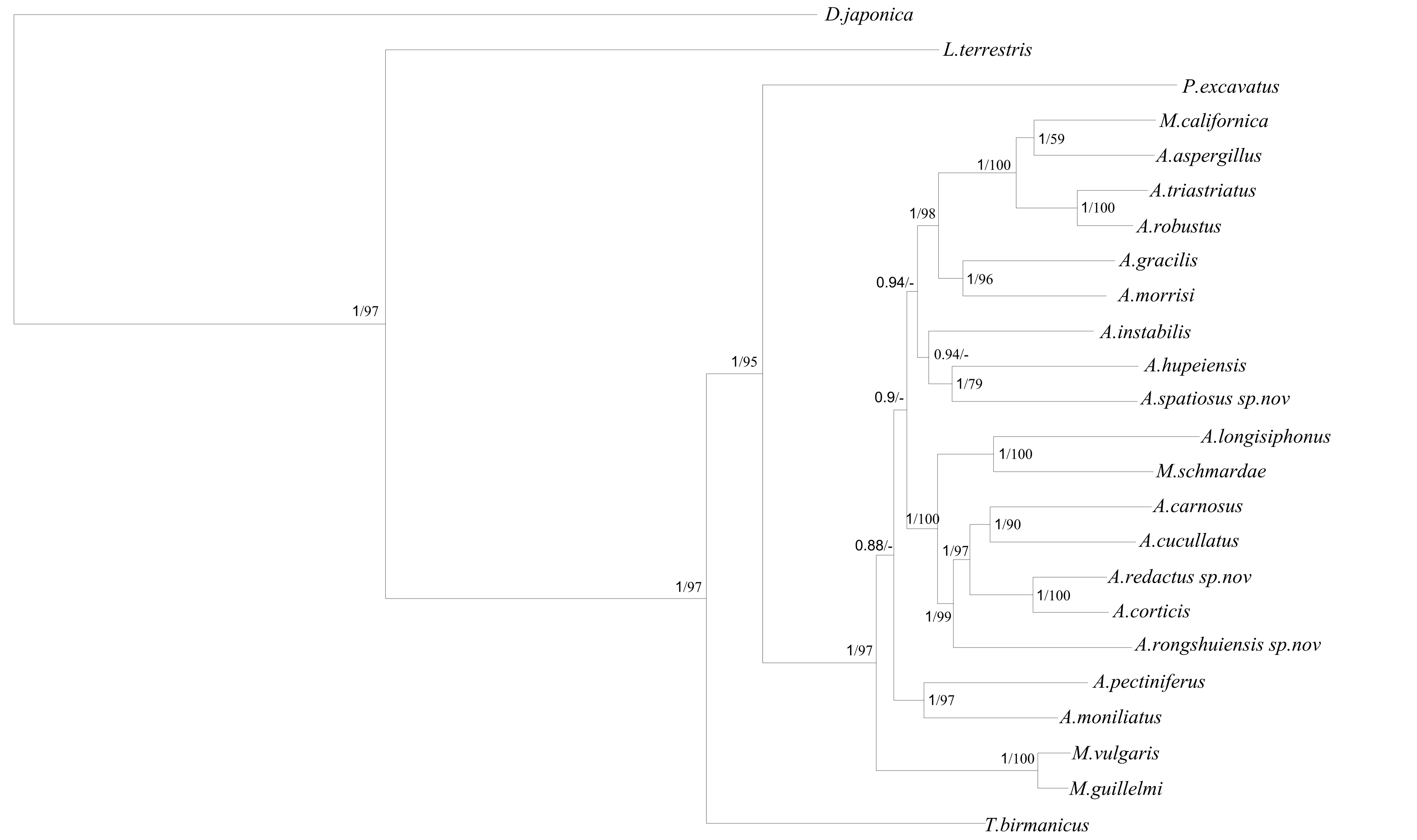

Supplementary Figure S2-5 The BI and ML tree inferred from the the PCGtri dataset

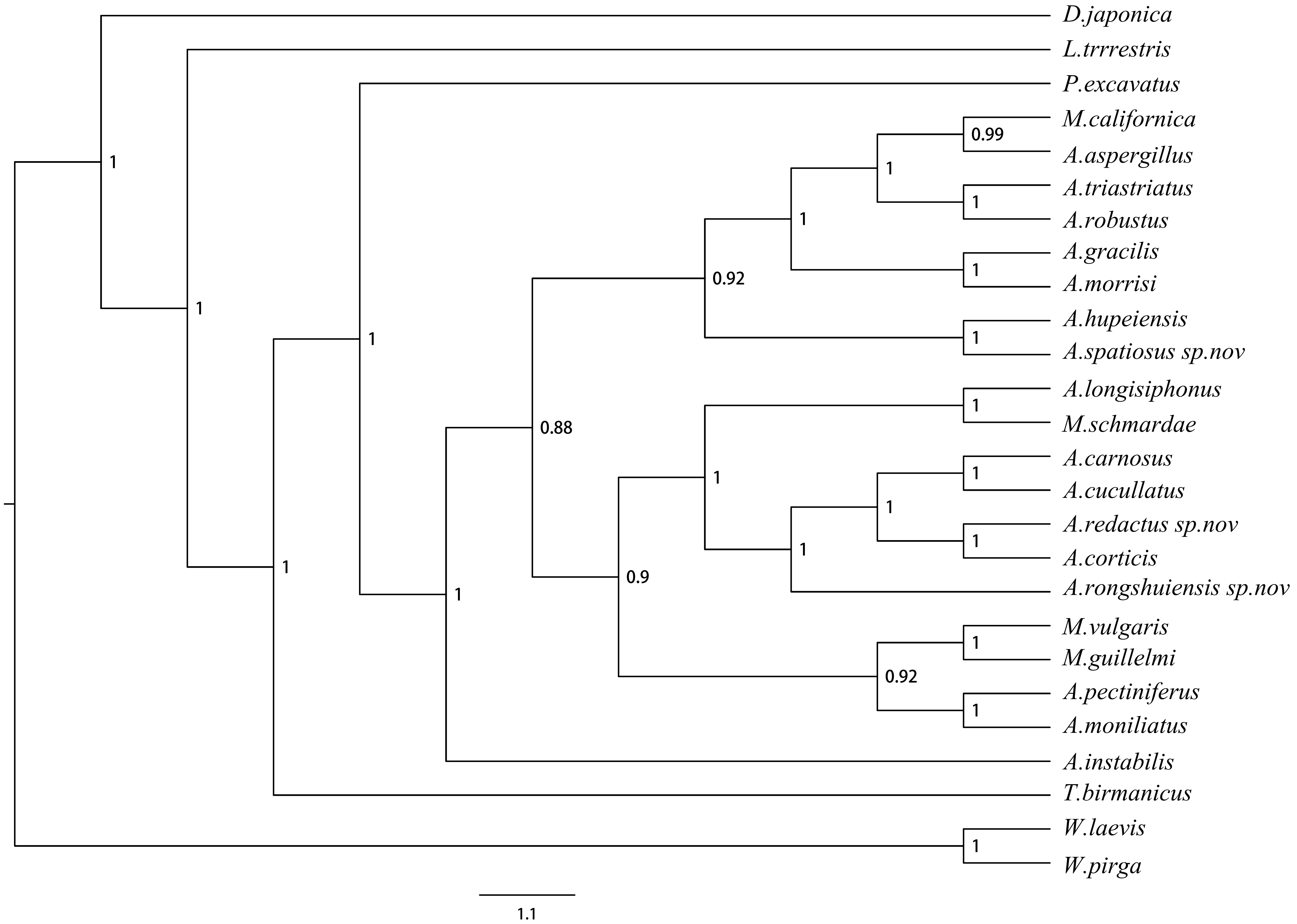

Supplementary Figure S2-6 The BI tree inferred from the the PT dataset

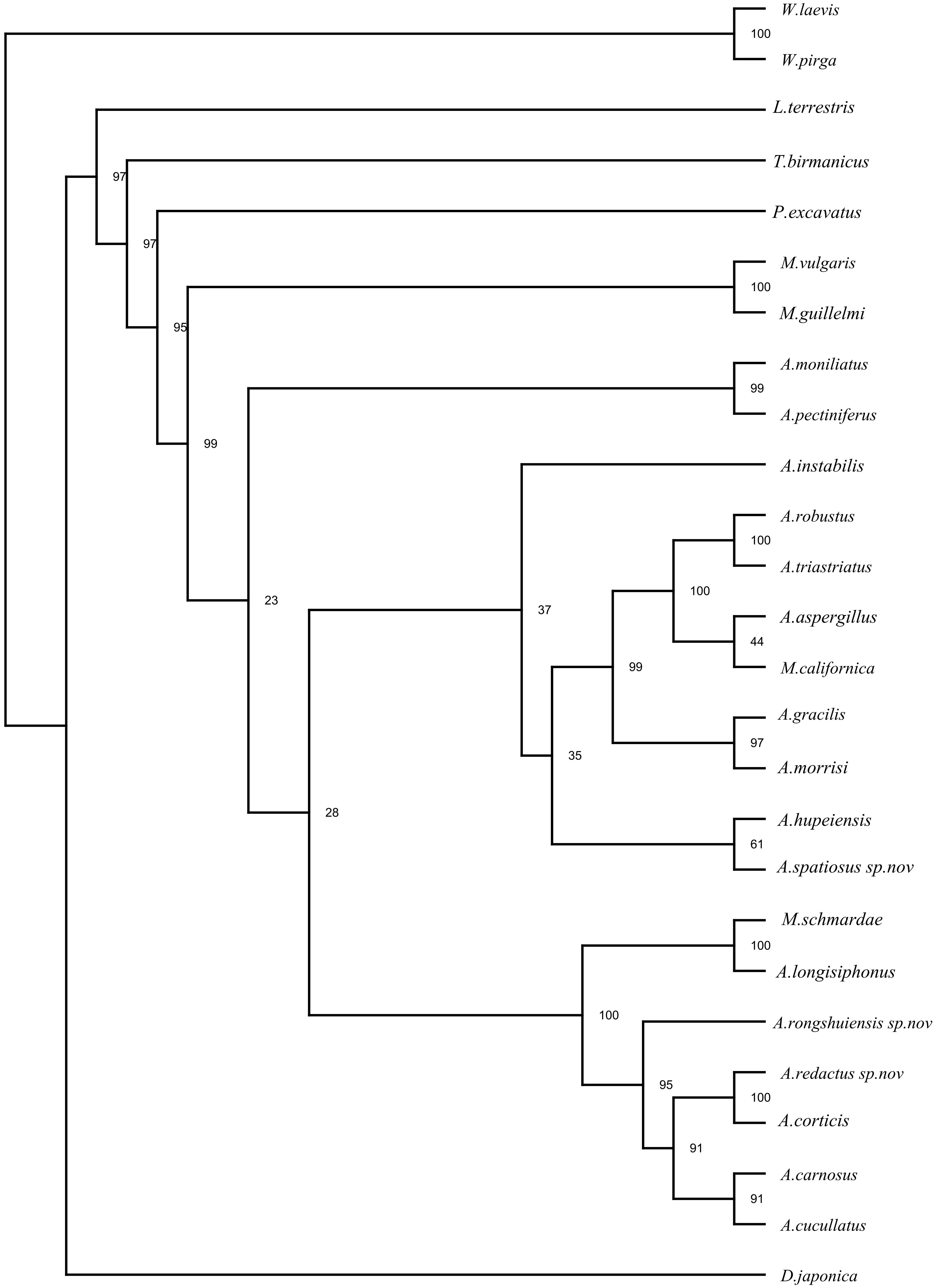

Supplementary Figure S2-7 The ML tree inferred from the the PT dataset

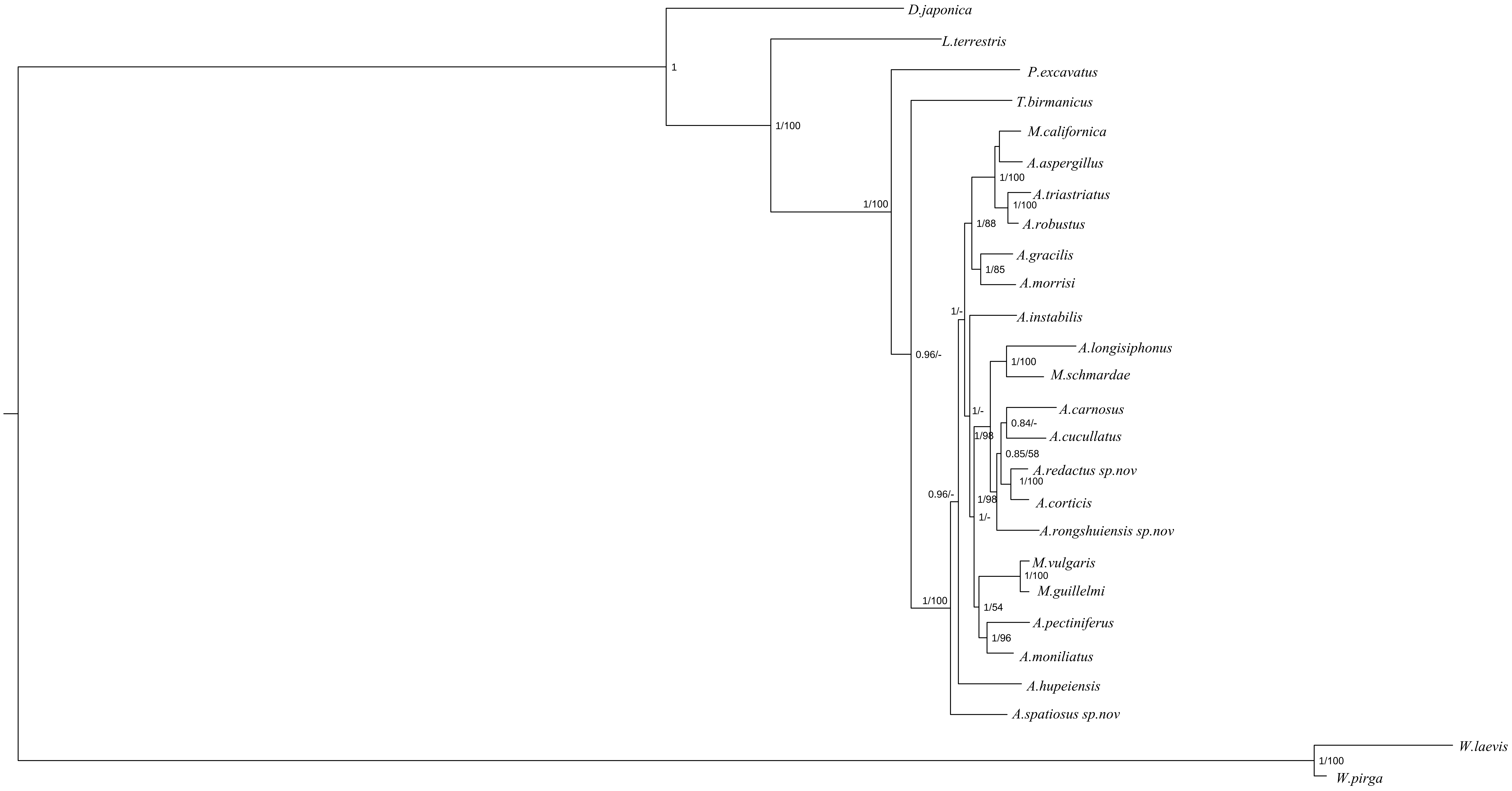

Supplementary Figure S2-8 The tree was inferred from the the PROgb dataset. Only bootstrap values more than 50% are shown and the others are represented by "-". The first numbers are from Bayesian inferences (PP) and the second numbers are from ML analyses (BP) .

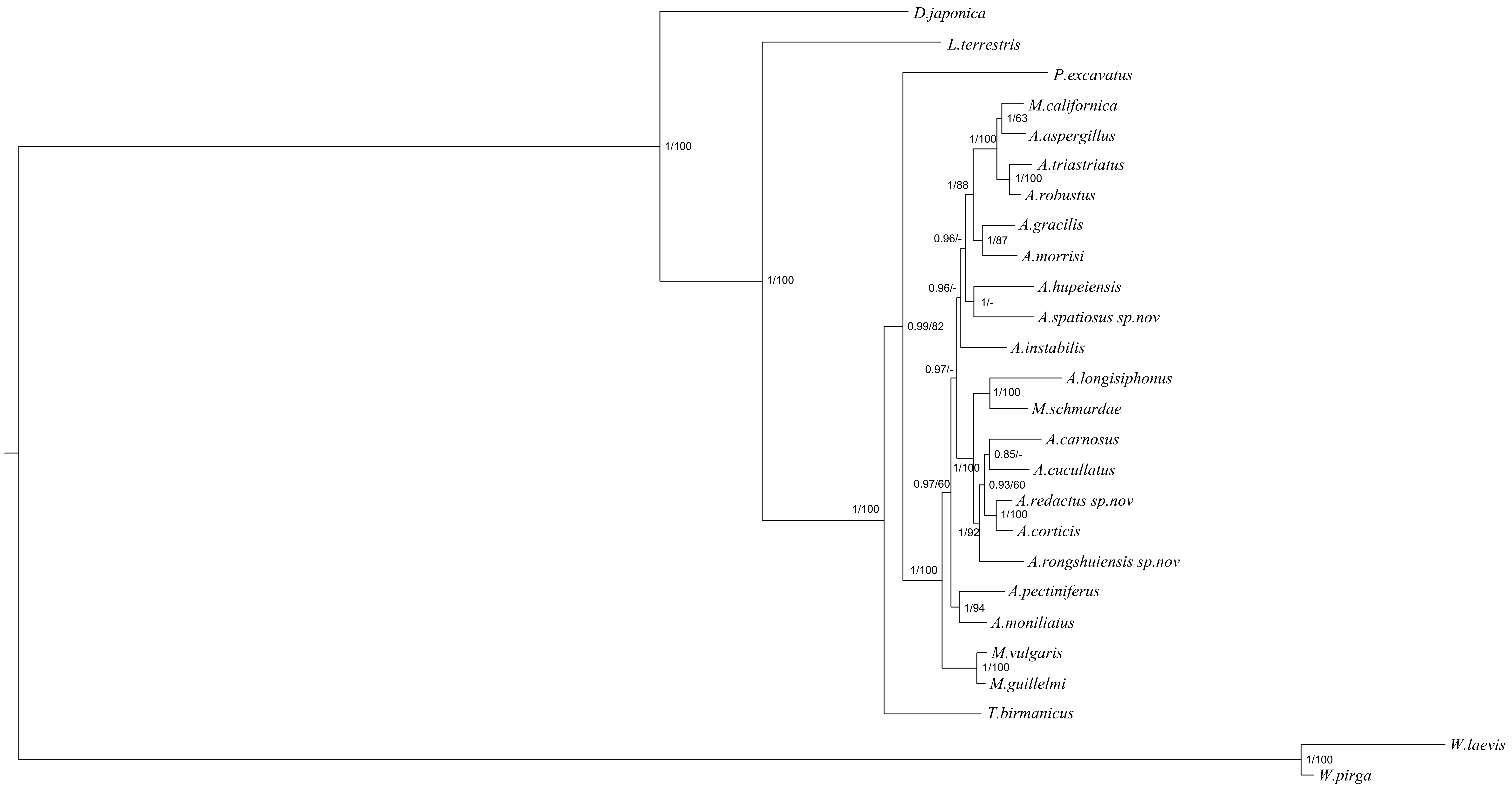

Supplementary Figure S2-9 The tree was inferred from the the PROtri dataset. Only bootstrap values more than 50% are shown and the others are represented by "-". The first numbers are from Bayesian inferences (PP) and the second numbers are from ML analyses (BP) .

Supplementary Figure S3 The BI tree inferred from the the PCGrRNAtri (PRtri) dataset

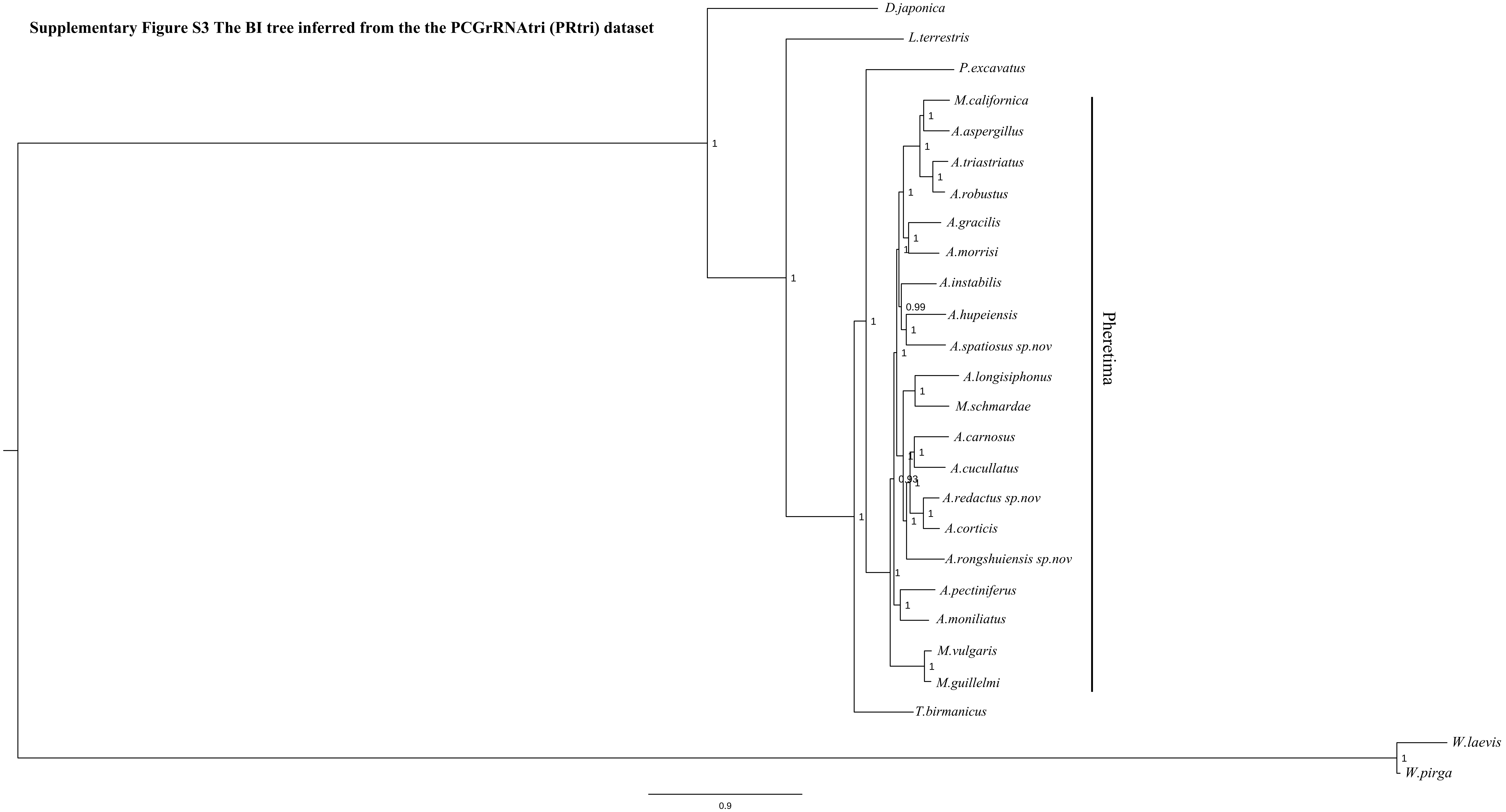

Supplementary Table S1 General Primers used in this study

General Primers	Sequences（5’-3’）	Target gene
CO1F	TTCWACAAATCATAAAGATATTGG	*CO1*
CO1R	TAWACTTCWGGRTGDCCAAAAARTCA	*CO1*
CO2F	ACHATYGGNCAYCARTGATAYTGA	*CO2*
CO2R	CCRCARATTTCDGARCAYTGNCC	*CO2*
CO3F	GTNACNGTHACHTGRGCHCAYCA	*CO3*
CO3R	TCDACRAARTGYCARTAYCADGC	*CO3*
CytbF	TTYTGRGGNGCDACNGTWATYAC	*Cytb*
CytbR	CCWAGYTTRTTHGGRATDGAHCG	*Cytb*
ND5F	AADGCCTTRAAYAAWGCRTGWGT	*ND5*
ND5R	GCDGCDATRGCHGCHCCTAC	*ND5*
ND4F	TGRGGNTAYCARCCDGARCG	*ND4*
ND4R	GGNGCYTCWACRTGDGCYTTHGG	*ND4*
16SF	ATYCTRACYGTGCAAAGGTAGC	*16S*
16SR	GGATAGAADCTAACCTGGCT	*16S*
ND1F	CGHAARGGNCCWAATAARGTWGG	*ND1*
ND1R	AGRTGRTCRTANCGYATDCGNGG	*ND1*

Supplementary Table S2 Specific primers used in this study

Species name	Specific primers
A.carnosus	CO1-CO2-F1, CTATTTGTATGAGCAGTAGT<br>CO1-CO2-R1, CGGTTGTCTACCTCTAGTAGGCGG<br>CO2-CO3-F1, GTACTKCACTCMTGAACAGTACC<br>CO2-CO3-R1, GTTCCGTATACCCTGTCTGC<br>CO3-CytB-F1, CGTGTGTTAATTGGGTCCAGG<br>CO3-CytB-R1, TAGGGTGGCATTATCTACTGC<br>CytB-ND5-F1, GCCCAACCTATTCACAGACCC<br>CytB-ND5-R1, CAGGGGGTTGATCTTATGAGCG<br>ND5-ND4-F1, GGGCAATAACTGAGTGTGAC<br>ND5-ND4-R1, GTGTTGTTTTCGAGTAGGCATAG<br>ND4-16S-F1, GGTTCCCAATAGATTACCCAAC<br>ND4-16S-R1, GCTCTATAGGGTCTTCTTGTC<br>16S-ND1-F1, GACAAGAAGACCCTATAGAGC<br>16S-ND1-R1, CGGGGATTTGTTAGCTGGTGTGGG<br>ND1-CO1-F1, GAATACCAAATATACCCATGTC<br>ND1-CO1-R1, TGTGCTGTTACAATTGTGTT
A.hupeiensis	CO1-CO2-F1, CTACACTTAGCAGGAGCATC<br>CO1-CO2-R1, GAGTGTAGYACATCTGCTGCAGT<br>CO2-CO3-F1, GGGGTTAAAGTAGATGCCGTACC<br>CO2-CO3-R1, GTTCCGTATACCCTGTCTGC<br>CO3-CytB-F1, GTATTAATTGGGTCAACGTTCC<br>CO3-CytB-R1, GAGAAGAATCGATTTAGGGTTGCG CytB-ND5-F1, CTTGTATAGTACTGTTTGAACCC<br>CytB-ND5-R1, CAGGTGGTTGATCTTAGAAATGG<br>ND5-ND4-F1, GCCCTACTATTCATCTGTGCTGGC<br>ND5-ND4-R1, GGGGCGTTTTGGAACATACATAG<br>ND4-ND1-F1, CCTTTCCAACAGATTACCCAGC<br>ND4-ND1-R1, GTTGGAGCTACTATAAATGGGGTC<br>ND1-CO1-F1, GAATACCAAATATACCCATGTC<br>ND1-CO1-R1, TGTGCTGTTACAATTGTGTT
M.guillelmi	CO1-CO2-F1, GTGTGGGCAGTAGTAATTACCG<br>CO1-CO2-R1, GGTACAACGATACGATTATCTACCTC<br>CO2-CO3-F1, GCCGATGTATTACACTCATG<br>CO2-CO3-R1, CTGCAATTGTAAAGGGGGCAGC<br>CO3-CytB-F1, GGACTACATGTACTAATTGGATCC<br>CO3-CytB-R1, AATGTGKAGAATTGTGGCCCC<br>CytB-ND5-F1, CCAAACCTATTTACCGACCCAG<br>CytB-ND5-R1, ATCAGGTGGTAGATCTTAGG<br>ND5-ND4-F1, GGGCCATAACAGAGTGTGAC<br>ND5-ND4-R1, CCYCCMAGWGTTATTAGTCATGC<br>ND4-16S-F1, TTTAYACAGTTACWGCCTCCCT<br>ND4-16S-R1, GCTCTATAGGGTCTTCTTGTC<br>16S-ND1-F1, GACAAGAAGACCCTATAGAGC<br>16S-ND1-R1, GGTAGGGGCAACTATGAATGGGGC<br>ND1-CO1-F1, CGACTTCGCTGAGGGGGAATCAG<br>ND1-CO1-R1, CCGGGCTGTCTTAGCTCAATTCG
A.pectiniferus	CO1-CO2-F1, CTATTTGTATGAGCAGTAGT<br>CO1-CO2-R1, CGGATTTCTAGTTGTATGGG<br>CO2-CO3-F1, CTAGGAGTTAAAGTAGATGC<br>CO2-CO3-R1, TTCCCCAGCTTGAAGTACAG<br>CO3-CytB-F1, CACTGGATTCCACGGATTACACG<br>CO3-CytB-R1, AATGTGKAGAATTGTGGCCCC

	CytB-ND5-F1, CCAAACCTTTTCACAGACCCT<br>CytB-ND5-R1, GGAGTTGATCTAAGAAAGGGG<br>ND5-ND4-F1, GCTCTATCTACATTAAGTCAGC<br>ND5-ND4-R1, GTGTGTTGTTTTTGAAGTCAG<br>ND4-16S-F1, AAGCTTCCCAACGGACTACCC<br>ND4-16S-R1, ATTCGGTTGGGGCGACCCAGGG<br>16S-ND1-F1, GCTCTATAGGGTCTTCTTGTCC<br>16S-ND1-R1, GTAGGGGCAACTATAAATGGGG<br>ND1-CO1-F1, GGGAGAATCCGAACTAGTGTCAGG<br>ND1-CO1-R1, GCCCAGGAATGAGCCCGGTTGTC
A.morrisi	CO1-CO2-F1, TACTAACAGACCGAAACC<br>CO1-CO2-R1, GTAATCGATAATCCCCCGG<br>CO2-CO3-F1, TAGGGGTYAAAGTAGATGC<br>CO2-CO3-R1, GCRGCMAGGTATTCACCAGC<br>CO3-CytB-F1, CCATGTACTAATTGGATCTAG<br>CO3-CytB-R1, CCGATTTAGGGTGGCATTGTC<br>CytB-ND5-F1, CCAGATGGAGCACCAGATTG<br>CytB-ND5-R1, CAGGTTGTTGATCTTAGG<br>ND5-ND4-F1, CTCTAAGACAGCTTGGAATG<br>ND5-ND4-R1, GTGGGTTGTTTTGGAACAC<br>ND4-16S-F1, CCCAATAGCGGCAATTGCG<br>ND4-16S-R1, GGGTCTTCTTGTCCTTCGAG<br>16S-ND1-F1, ATTCGGTTGGGGCGACCC<br>16S-ND1-R1, GGGGGCAACTATGAATGGAGAC<br>ND1-CO1-F1, GCCCAACATTTTAGTATCCG<br>ND1-CO1-R1, GGTTGTCTTAGTTCAATTCG
A.robustus	CO1-CO2-F1, TACTAACAGATCGAAACC<br>CO1-CO2-R1, CTTCTAGTAGTCGAAAGTC<br>CO2-CO3-F1, AAGTAGAYGCCGTACCTGG<br>CO2-CO3-R1, CCCTRTCTGCAATTGTAAATGG<br>CO3-CytB-F1, TTAATTGGATCTAGATTCC<br>CO3-CytB-R1, CCAATTGGGTTATTAGATCCTG<br>CytB-ND5-F1, CCTAACCTATTTACAGATCCTG<br>CytB-ND5-R1, CATCTCCAATTCGGTTTG<br>ND5-ND4-F1, CAACRCTAAGTCAACTTGG<br>ND5-ND4-R1, GGTATATGTGTTGTTTTAGAAG<br>ND4-16S-F1, CCAGCTGCAACAATTGCATGAC<br>ND4-16S-R1, GGGTCTTCTTGTCCTTCGAG<br>16S-ND1-F1, ATTCGGTTGGGGCGACCC<br>16S-ND1-R1, AGGGCTATAGTTGGGGCA<br>ND1-CO1-F1, ATCTGACACTATACTTCTC<br>ND1-CO1-R1, AATGCATGTGCTGTTACA
A.triastriatus	CO1-CO2-F1, TACTAACAGATCGAAACC<br>CO1-CO2-R1, CTTCTAGTAGTCGAAAGTC<br>CO2-CO3-F1, AAGTAGAYGCCGTACCTGG<br>CO2-CO3-R1, CCCTRTCTGCAATTGTAAATGG<br>CO3-CytB-F1, TTAATTGGATCTAGATTCC<br>CO3-CytB-R1, GAATCGGTTTAGGGTAGC<br>CytB-ND5-F1, GGGCATAGTGCCCTACAC<br>CytB-ND5-R1, CATCTCCAATTCGGTTTG<br>ND5-ND4-F1, CAACRCTAAGTCAACTTGG<br>ND5-ND4-R1, GGTATGTGAGTTGTTTTAGAAG<br>ND4-16S-F1, CAGACTTCCCTGCGGCAAC<br>ND4-16S-R1, GGGTCTTCTTGTCCTTCGAG<br>16S-ND1-F1, ATTCGGTTGGGGCGACCC<br>16S-ND1-R1, AGGGCTATAGTTGGGGCA

	ND1-CO1-F1, CGTATTCATAAGAGTACCAAG<br>ND1-CO1-R1, AATGCATGTGCTGTTACA
A.instabilis	CO1-CO2-F1, CTATTAACAGACCGCAATC<br>CO1-CO2-R1, GACAATTCGATTATCTAC<br>CO2-CO3-F1, TAGGGGTAAAAGTAGATGC<br>CO2-CO3-R1, GCRGCMAGGTATTCACCAGC<br>CO3-CytB-F1, CACGTACTAATCGGATCTAC<br>CO3-CytB-R1, GAGTAGCATTATCTACTGC<br>CytB-ND5-F1, AACCTATTCACAGACCCAG<br>CytB-ND5-R1, ATWGTGGTTGTAGATACTGC<br>ND5-ND4-F1, GCYCTATCAACACTAAGAC<br>ND5-ND4-R1, GTGTTGTCTTAGAACATCTG<br>ND4-16S-F1, CCCAGCAGTTACAGTTGCG<br>ND4-16S-R1, GGGTCTTCTTGTCCTTCGAG<br>16S-ND1-F1, ATTCGGTTGGGGCGACCC<br>16S-ND1-R1, GCTAARATTAGGGCTATGG<br>ND1-CO1-F1, CCAAACATATTTATATCAGACAC<br>ND1-CO1-R1, GGAATGAGCCGGGCTGTC
M.schmardae	CO1-CO2-F1, ATTRACAGATCGAAACCT<br>CO1-CO2-R1, AACGATTCGGTTATCTAC<br>CO2-CO3-F1, TAGGGGTYAAAGTAGATGC<br>CO2-CO3-R1, CCATATACTCTATCTGCAA<br>CO3-CytB-F1, CCTTCTTTGTAGCCACTGG<br>CO3-CytB-R1, ATGTGGCGTTATCCACTGC<br>CytB-ND5-F1, GGGTTCCCCATATGGCTGAG<br>CytB-ND5-R1, AGTTGATCTTAGAAATGG<br>ND5-ND4-F1, CTACAYTAAGACAACTAGG<br>ND5-ND4-R1, GTGAAGTTTTAGAGGACATG<br>ND4-16S-F1, CCCAACAACCACACTGGC<br>ND4-16S-R1, GGGTCTTCTTGTCCTTCGAG<br>16S-ND1-F1, ATTCGGTTGGGGCGACCC<br>16S-ND1-R1, AGGGCTATAGTTGGGGCA<br>ND1-CO1-F1, GCCAAACATAGCCCTATCAG<br>ND1-CO1-R1, GGCTGTCTTAGYTCAATTCG
A.cucullatus	CO1-CO2-F1, TACTAACAGATCGAAACC<br>CO1-CO2-R1, GACAATTCGATTATCTAC<br>CO2-CO3-F1, TAGGGGTAAAAGTAGATGC<br>CO2-CO3-R1, CCCTRTCTGCAATTGTAAATGG<br>CO3-CytB-F1, GTAGCTACCGGATTTCACGG<br>CO3-CytB-R1, GGTGGCGCTATTTACTGC<br>CytB-ND5-F1, CCGACCCAGAAAACTTCC<br>CytB-ND5-R1, GGTAGTTGATCTTAGAAATGGG<br>ND5-ND4-F1, CAACACTAAGTCAACTAGG<br>ND5-ND4-R1, GTGTTGTTTTTGAAGTTGC<br>ND4-16S-F1, CCCAGCCGTTACAATCGC<br>ND4-16S-R1, GGGTCTTCTTGTCCTTCGAG<br>16S-ND1-F1, ATTCGGTTGGGGCGACCC<br>16S-ND1-R1, GCTAARATTAGGGCTATGG<br>ND1-CO1-F1, CCCAACATACTTATATCTG<br>ND1-CO1-R1, GGCTGTCTTAGYTCAATTCG
A.redactus sp.nov	CO1-CO2-F1, TACTAACAGATCGAAACC<br>CO1-CO2-R1, GTCGGAAGTCACCTGGGG<br>CO2-CO3-F1, GTTAAAGTAGATGCTGTGCC<br>CO2-CO3-R1, CCATATACTCTATCTGCAA<br>CO3-CytB-F1, GTAGCTACCGGATTTCACGG<br>CO3-CytB-R1, CCAATTGGGTTATTAGATCCTG

	CytB-ND5-F1, CCTAACCTATTTACAGATCCTG<br>CytB-ND5-R1, CAGGTTGTTGATCTTAGG<br>ND5-ND4-F1, CTCTAAGACAGCTTGGAATG<br>ND5-ND4-R1, GAGTTGTTTTTGAACACC<br>ND4-16S-F1, CCCGACAGTAACAATTGC<br>ND4-16S-R1, GGGTCTTCTTGTCCTTCGAG<br>16S-ND1-F1, ATTCGGTTGGGGCGACCC<br>16S-ND1-R1, GGTGATTTATTGGCTGGTGTGGG<br>ND1-CO1-F1, CCAAACATATTTATGTCAG<br>ND1-CO1-R1, AATGCATGTGCTGTTACA
A.moniliatus	CO1-CO2-F1, TACTAACAGACCGAAACC<br>CO1-CO2-R1, CTTCTAAGAGACGGAAATCTC<br>CO2-CO3-F1, TMTTACACTCATGAACTGT<br>CO2-CO3-R1, CCCTRTCTGCAATTGTAAATGG<br>CO3-CytB-F1, GTAGCTACCGGATTTCACGG<br>CO3-CytB-R1, GGGTTGCGTTATCTACTGC<br>CytB-ND5-F1, GAGCCAAACCTATTTACCG<br>CytB-ND5-R1, AGTTGATCTTAGAAATGG<br>ND5-ND4-F1, CAACTCTAAGACAGCTTGG<br>ND5-ND4-R1, GGTATATGTGTTGTTTTAGAACAC<br>ND4-16S-F1, GCAGTAACAATTGCATGAC<br>ND4-16S-R1, GGGTCTTCTTGTCCTTCGAG<br>16S-ND1-F1, ATTCGGTTGGGGCGACCC<br>16S-ND1-R1, GGAGATTTGTTTGCCGGCG<br>ND1-CO1-F1, CAGACATAATGCTTCTAC<br>ND1-CO1-R1, GGTTGTCTTAGTTCAATTCG
A.spatiosus sp.nov	CO1-CO2-F1, ATTRACAGATCGAAACCT<br>CO1-CO2-R1, AACGATTCGGTTATCTAC<br>CO2-CO3-F1, TAGGGGTYAAAGTAGATGC<br>CO2-CO3-R1, TAGGGGTYAAAGTAGATGC<br>CO3-CytB-F1, CATGTACTAATTGGATCCAG<br>CO3-CytB-R1, CCGATTAAGTGTGGCGCTACC<br>CytB-ND5-F1, GTWCTATTTGAACCAAACCT<br>CytB-ND5-R1, ATWGTGGTTGTAGATACTGC<br>ND5-ND4-F1, GCYCTATCAACACTAAGAC<br>ND5-ND4-R1, GGGTTGTTTTGGAGGCTAGG<br>ND4-16S-F1, CCCAGTAATCACAATTGC<br>ND4-16S-R1, GGGTCTTCTTGTCCTTCGAG<br>16S-ND1-F1, ATTCGGTTGGGGCGACCC<br>16S-ND1-R1, GGGGCTACTATGAAAGGTGAC<br>ND1-CO1-F1, GTATCATCTTTATAAGGATACC<br>ND1-CO1-R1, AATGCATGTGCTGTTACA
A.rongshuiensis sp.nov	CO1-CO2-F1, CTACTGACAGATCGAAACC<br>CO1-CO2-R1, CTTCCAGGAGACGAAAGTC<br>CO2-CO3-F1, CAGTGCCATCTCTTGGGG<br>CO2-CO3-R1, CGTATTCCCCGGCCTGAAG<br>CO3-CytB-F1, GGATTTCACGGTCTTCATG<br>CO3-CytB-R1, GAATCGATTTAAGGTGGCG<br>CytB-ND5-F1, CACCCTTGTACTCGGTTTCCC<br>CytB-ND5-R1, GGATGGTCATGAAAAAGAG<br>ND5-ND4-F1, GCCCTATCAACACTAAGAC<br>ND5-ND4-R1, GCATAGAGCTACTAATATTGG<br>ND4-16S-F1, CCAATAGACTACCCAGCAG<br>ND4-16S-R1, GGGTCTTCTTGTCCTTCGAG<br>16S-ND1-F1, ATTCGGTTGGGGCGACCC<br>16S-ND1-R1, GGGGATTTGTTGGCGGGTG

	ND1-CO1-F1, TTCATAAGAATACCCAAC<br>ND1-CO1-R1, GGAATGACCCGGGTTGTC
D.japonica	CO1-CO2-F1, ATTRACAGATCGAAACCT<br>CO1-CO2-R1, AACGATTCGGTTATCTAC<br>CO2-CO3-F1, TAGGGGTYAAAGTAGATGC<br>CO2-CO3-R1, CACCTGCTTGAAGAAAGG<br>CO3-CytB-F1, CATGTACTAATTGGATCCAG<br>CO3-CytB-R1, CCGATTAAGTGTGGCGCTACC<br>CytB-ND5-F1, GTWCTATTTGAACCAAACCT<br>CytB-ND5-R1, ATWGTGGTTGTAGATACTGC<br>ND5-ND4-F1, GCYCTATCAACACTAAGAC<br>ND5-ND4-R1, GGGTTGTTTTGGAGGCTAGG<br>ND4-16S-F1, CCCAGTAATCACAATTGC<br>ND4-16S-R1, GGGTCTTCTTGTCCTTCGAG<br>16S-ND1-F1, ATTCGGTTGGGGCGACCC<br>16S-ND1-R1, GGGGCTACTATGAAAGGTGAC<br>ND1-CO1-F1, GTATCATCTTTATAAGGATACC<br>ND1-CO1-R1, AATGCATGTGCTGTTACA

Supplementary Table S3 Mean P distances for 24 earthworms based on 13 PCGs.

		[1]	[2]	[3]	[4]	[5]	[6]	[7]	[8]	[9]	[10]	[11]	[12]	[13]	[14]	[15]	[16]	[17]	[18]	[19]	[20]	[21]	[22]	[23]	[24]
[1]	*A.aspergillus*																								
[2]	*A.carnosus*	0.215																							
[3]	*A.corticis*	0.198	0.177																						
[4]	*A.cucullatus*	0.212	0.186	0.174																					
[5]	*A.gracilis*	0.193	0.204	0.190	0.193																				
[6]	*A.hupeiensis*	0.205	0.211	0.196	0.204	0.193																			
[7]	*A.instabilis*	0.200	0.200	0.187	0.197	0.188	0.193																		
[8]	*A.longisiphonus*	0.219	0.216	0.196	0.205	0.209	0.215	0.210																	
[9]	*A.moniliatus*	0.195	0.200	0.180	0.189	0.183	0.181	0.182	0.199																
[10]	*A.morrisi*	0.195	0.206	0.185	0.194	0.172	0.193	0.185	0.211	0.181															
[11]	*A.pectiniferus*	0.198	0.209	0.187	0.201	0.190	0.197	0.194	0.205	0.172	0.192														
[12]	*A.redactus_sp.nov*	0.195	0.179	0.111	0.167	0.180	0.192	0.177	0.189	0.171	0.179	0.179													
[13]	*A.robustus*	0.162	0.210	0.199	0.200	0.187	0.196	0.193	0.214	0.190	0.188	0.199	0.191												
[14]	*A.rongshuiensis_sp.nov*	0.207	0.195	0.168	0.182	0.191	0.199	0.193	0.200	0.181	0.195	0.193	0.169	0.201											
[15]	*A.spatiosus_sp.nov*	0.207	0.209	0.193	0.203	0.193	0.194	0.189	0.217	0.186	0.194	0.196	0.185	0.199	0.197										
[16]	*A.triastriatus*	0.167	0.213	0.195	0.202	0.185	0.200	0.197	0.219	0.192	0.193	0.199	0.194	0.108	0.202	0.198									
[17]	*L.terrestris*	0.314	0.313	0.308	0.311	0.302	0.305	0.307	0.312	0.299	0.306	0.305	0.304	0.308	0.307	0.307	0.313								
[18]	*M.californica*	0.160	0.213	0.201	0.206	0.187	0.200	0.195	0.221	0.191	0.190	0.201	0.192	0.160	0.208	0.199	0.166	0.305							
[19]	*M.guillelmi*	0.209	0.210	0.198	0.203	0.194	0.207	0.191	0.214	0.185	0.196	0.197	0.193	0.202	0.198	0.203	0.204	0.306	0.203						
[20]	*M.schmardae*	0.215	0.205	0.184	0.193	0.199	0.204	0.202	0.192	0.189	0.202	0.200	0.182	0.205	0.185	0.203	0.205	0.308	0.208	0.206					
[21]	*M.vulgaris*	0.210	0.213	0.198	0.204	0.195	0.208	0.194	0.216	0.186	0.198	0.199	0.194	0.202	0.202	0.204	0.203	0.309	0.204	0.062	0.207				
[22]	*P.excavatus*	0.261	0.267	0.253	0.252	0.253	0.251	0.252	0.260	0.243	0.249	0.253	0.245	0.258	0.249	0.249	0.256	0.310	0.258	0.254	0.254	0.257			
[23]	*T.birmanicus*	0.245	0.250	0.242	0.249	0.242	0.247	0.241	0.257	0.231	0.243	0.241	0.238	0.248	0.242	0.242	0.247	0.310	0.245	0.249	0.246	0.249	0.263		
[24]	*D.japonica*	0.344	0.346	0.330	0.339	0.329	0.331	0.337	0.333	0.324	0.328	0.332	0.325	0.332	0.322	0.330	0.336	0.343	0.341	0.335	0.330	0.336	0.341	0.343	

Supplementary Table S4 Summary of multiple alignments of tRNA genes in earthworm mtDNAs.

**ALN**	**amino acid**	**alignment length**	**identical positions**	**%INUC**
trnA	Ala	65	37	56.92
trnC	Cys	68	35	51.47
trnD	Asp	67	35	52.24
trnE	Glu	70	39	55.71
trnF	Phe	66	28	42.42
trnG	Gly	68	39	57.35
trnH	His	67	35	52.24
trnI	Ile	66	41	62.12
trnK	Lys	66	42	63.64
trnL1	Leu (CUN)	65	28	43.08
trnL2	Leu (UUR)	65	37	56.92
trnM	Met	64	45	70.31
trnN	Asn	67	45	67.16
trnP	Pro	67	33	49.25
trnQ	Gln	70	38	54.29
trnR	Arg	66	39	59.09
trnS1	Ser (AGN)	64	32	50.00
trnS2	Ser (UCN)	67	25	37.31
trnT	Thr	68	35	51.47
trnV	Val	67	38	56.72
trnW	Trp	66	39	59.09
trnY	Tyr	67	35	52.24

ALN, alignment name; %INUC, percent of identical nucleotides
